# Depression, Anxiety, Emotional Eating, and Body Mass Index among Self-Reported Vegetarians and Non-Vegetarians: A Cross-Sectional Study in Peruvian Adults

**DOI:** 10.3390/nu16111663

**Published:** 2024-05-29

**Authors:** Jacksaint Saintila, Sandra P. Carranza-Cubas, Antonio Serpa-Barrientos, Renzo Felipe Carranza Esteban, Denis Frank Cunza-Aranzábal, Yaquelin E. Calizaya-Milla

**Affiliations:** 1 School of Medicine, Señor de Sipan University, Chiclayo 14001, Peru; ccubassandrapao@uss.edu.pe; 2 Department of Psychology, National University of San Marcos, Lima 15457, Peru; aserpab@unmsm.edu.pe; 3 Grupo de Investigación Avances en Investigación Psicológica, Facultad de Ciencias de la Salud, Universidad San Ignacio de Loyola, Lima 15024, Peru; rcarranza@usil.edu.pe; 4 School of Psychology, Peruvian Union University, Tarapoto 22201, Peru; deniscunza@upeu.edu.pe; 5 Research Group for Nutrition and Lifestyle, Peruvian Union University, Lima 15457, Peru

**Keywords:** anxiety, body mass index, depression, emotional eating, dietary pattern, vegetarians

## Abstract

**Background**: Vegetarianism is commonly associated with various health benefits. However, the association between this dietary regimen and aspects of mental health remains ambiguous. This study compared the symptoms of depression and anxiety, emotional eating (EmE), and body mass index (BMI) in Peruvian vegetarian and non-vegetarian adults. **Methods**: A cross-sectional study was conducted on 768 Peruvian adults, of whom 284 (37%) were vegetarians and 484 (63%) were non-vegetarians. The Depression Patient Health Questionnaire-2 (PHQ-2), Generalized Anxiety Disorder Scale-2 (GAD-2), and an EmE questionnaire were applied; additionally, the BMI was calculated. Simple and multiple linear regression and Poisson regression models with robust variance were used to evaluate the association between depression, anxiety, EmE, and BMI with dietary patterns. **Results**: The vegetarians (Adjusted Prevalence Ratio [PR] = 0.24, 95% CI 0.16–0.31; *p* < 0.001) reported more depressive symptoms than the non-vegetarians. This trend persisted for anxiety, with an adjusted PR of 0.17 (95% CI: 0.01–0.29; *p* = 0.012). However, the vegetarians (adjusted PR = −0.38, 95% CI: −0.61–−0.14; *p* < 0.001) reported lower EmE scores compared to the non-vegetarians. Likewise, the vegetarians had a lower mean BMI than the non-vegetarians (B = −0.16, 95% CI: −0.21–−0.08; *p* < 0.001). **Conclusions**: Vegetarian diets are associated with increased symptoms of depression and anxiety, as well as lower EmE and BMI scores. Further longitudinal studies are needed to elucidate these associations and determine causality and the underlying mechanisms involved.

## 1. Introduction

Vegetarianism, characterized by a dietary pattern that excludes mainly red meat and, in many cases, also poultry, fish, and seafood, is recognized by its variety and diversity [1]. Among these varieties are lacto-vegetarianism, which allows the consumption of dairy products; ovo-vegetarianism, which includes eggs; and lacto-ovo-vegetarianism, which allows both eggs and dairy products [1]. In addition, flexitarianism or semi-vegetarianism represents a more flexible approach, limiting meat consumption rather than excluding it completely [2]. Vegetarianism includes veganism, which is a stricter form that excludes all animal products, including dairy, eggs, honey, and animal derivatives in processed foods and other products [3]. Each of these dietary practices offers potential health benefits, including a reduced risk of noncommunicable diseases such as heart disease, type 2 diabetes, and certain types of cancer [2]. The health benefits of reducing or eliminating meat consumption can be attributed to a higher intake of fiber, polyunsaturated fats, vitamin C, and bioactive substances, as well as a lower intake of saturated fats [4]. However, the relationship between vegetarianism and individuals’ mental health remains a topic of debate and varying analyses.

Although vegetarian diets are recognized for their nutritional benefits and research suggests that they align more closely with public health recommendations than omnivorous diets, they present specific nutritional challenges [5]. In fact, some studies report possible deficiencies in critical nutrients, such as vitamin B-12, omega-3 fatty acids, folates, and zinc, which can negatively impact a person’s mental health [6,7,8].

Depression and anxiety are common mental disorders and rank among the top 25 causes of morbidity and disability worldwide [9]. In Peru, according to the Peruvian Ministry of Health, the cases of mental health conditions increased by almost 20% in 2022 [10]. In 2023, 80% of the Peruvian population experienced high levels of stress, anxiety, and depression due to the growing perception of citizen insecurity [10]. Both the onset and management of depression and anxiety can be influenced by diet. For example, Paslakis et al. [7] conducted a study in Germany which found that individuals who followed a vegetarian diet had higher rates of depression than their omnivorous counterparts. On the other hand, a study conducted by Matta et al. [11] with 90,380 French participants found that individuals who avoided eating meat showed a higher prevalence of depression symptoms (28.4%) compared to meat eaters (16.2%). However, this study found no association between a vegetarian diet and depressive symptoms among people with a high consumption of legumes. There is no consensus on the relationship between a vegetarian diet and depression or anxiety. For example, certain studies have failed to confirm an elevated risk of depression or anxiety among vegetarians [12]. In fact, other studies have reported reduced levels of anxiety and depression among individuals who follow a vegetarian diet [13]. This implies that the association between vegetarian dietary patterns and mental health may be more intricate than originally believed, potentially influenced by various dietary and non-dietary factors.

On the other hand, emotional eating is a maladaptive behavior characterized by excessive food consumption in response to negative emotions such as stress, anxiety, frustration, sadness, anger, and loneliness [14]. This behavior reflects a coping mechanism in which food intake is used to alleviate or manage adverse emotional states [15]. Research has demonstrated that negative emotions, such as emotional stress, can affect appetite and food consumption. Specifically, approximately 30% of people experience an increase in appetite or food consumption, while 48% experience a decrease in these aspects [16]. In relation to the connection between vegetarian diets and emotional eating, a cross-sectional survey of young women in Poland discovered that vegetarians scored lower on subscales of emotional eating [17]. Similarly, another study showed that vegans were less prone to emotional eating [18]. However, there are few studies that analyze both variables, and most of them focus on eating disorders [1,7].

Body mass index (BMI) is a valuable tool to assess weight status and the potential risk of developing health problems, such as obesity [19]. In fact, obesity represents one of the most important public health problems worldwide. In Peru, 37.5% of people aged 15 years and older are overweight, while 25.6% are obese [20]. Numerous scientific studies have focused on the relationship between BMI and vegetarian diets, reflecting a trend toward a lower BMI and reduced obesity rates among people who follow plant-based diets [2,3,21]. As the global population faces rising rates of obesity and related diseases, adopting well-planned vegetarian diets can be a valuable strategy for improving public health.

Although the existing literature provides valuable information on the relationship between vegetarian diets, depression, anxiety, and BMI, there are still notable limitations that justify the need for more specific research. Specifically, the relationship between vegetarian diets and mental health conditions, such as depression and anxiety, remains a topic of debate due to mixed and often contradictory results in studies [1,7,22]. It also highlights a distinct lack of studies exploring emotional eating in the context of vegetarian diets, leaving a gap in the understanding of how these dietary patterns may affect or be affected by emotional responses to eating. Therefore, there is a need to further investigate the association between these dietary aspects and mental and physical well-being, as these findings may lead to the development of more specific guidelines for health professionals for the follow-up of patients following this type of diet. This study aimed to compare depression, anxiety, EmE, and BMI between Peruvian vegetarian and non-vegetarian adults.

## 2. Materials and Methods

### 2.1. Design and Participants

An analytical observational study of cross-sectional design was conducted during the period October to December 2023 using an online survey. The nonprobability convenience sampling method was used to collect data from the participants. The sample size was determined using Soper’s Free Statistical Calculator version 4.0 [23], where considering a multiple regression analysis, an effect size of 0.10, a statistical power of 0.80, five explanatory variables, and a significance level of 0.05 were taken into account. According to these parameters, a minimum of 124 participants were estimated to be necessary for the sample. However, 801 participants volunteered for this study, of whom 768 were included in statistical analyses, exceeding the calculated sample size.

To contact participants, we used personal and institutional email addresses, as well as social media platforms such as WhatsApp and Facebook. Participants accessed and completed the questionnaire through the Google Forms platform. This study included participants aged 18 to 65 years. Those with prior diagnoses of severe psychiatric disorders (e.g., psychotic disorders, bipolar disorders), foreign residents, those under 18 years and older than 65 years, pregnant women, and those who did not give informed consent were excluded from this study.

### 2.2. Ethical Aspects

On the survey’s homepage, participants were informed about the study objectives. Participation was anonymous and voluntary. Informed consent was obtained from all participants. The study protocol was reviewed and approved by the Research Ethics Committee of the Faculty of Health Sciences of the Universidad Peruana Unión (Reference: 043-2023/UPeU/FCS, 22 September 2023). This study was carried out following the ethical principles for medical research outlined in the Declaration of Helsinki.

### 2.3. Variables and Instruments

Three self-reported instruments were used in this study. The first instrument measured depressive symptoms, the second measured anxiety symptoms, and the last measured emotional eating. The survey participants provided information on their sociodemographic characteristics, including age, sex, place of residence, religion, marital status, level of education, and monthly family income.

- Depression Patient Health Questionnarie-2 (PHQ-2)

This scale comprises the first two items of PHQ-9, which are fundamental criteria for depressive disorders. These elements are as follows: (1) feeling down, depressed, or hopeless and (2) little interest or pleasure in doing things [24]. It is composed of a Likert-type response scale, where “*No day*” = 0, “*Several days*” = 1, “*More than half of the days*” = 2, and “*Almost every day*” = 3. According to this scale, scores of 3 or higher suggest the presence of depression, while scores below 3 suggest the absence of depressive symptoms [25,26]. In the present study, the PHQ-2 showed adequate reliability (α = 0.72).

- Generalized Anxiety Disorder Scale-2 (GAD-2)

This is a scale composed of two items from GAD-7: item 1, “Feeling nervous, anxious or on edge”, and item 2, “Not being able to stop or control worrying”. Both are evaluated through the following statement: “Please indicate how often you have suffered the following problems in the last 15 days” [27]. Response options are Likert-type, where “*Never* = 0” and “*Almost every day* = 3” [27]. A score ≥ 3 is an indicator of a probable clinically relevant anxiety disorder, while a score < 3 indicates absence of anxiety symptoms. The internal consistency value obtained for this study is α = 0.84.

- Emotional Eating Questionnaire

This questionnaire is composed of a total of 10 questions and uses a Likert-type scale with four response options: “*Never* = 0”, “*Sometimes* = 1”, “*Usually* = 2”, and “*Always* = 3”. The sum of the scores obtained in each item allows participants to be classified into three categories: non-EmE (from 0 to 5 points), moderately EmE (from 6 to 10 points), and emotional or very EmE (from 11 to 30 points) [28]. In the present study, the questionnaire showed adequate reliability (α = 0.75).

- BMI

Participants self-reported weight and height. Subsequently, the body mass index (BMI) was calculated. WHO guidelines were taken into account to classify the BMI as “underweight” when it was <18.5; “normopeso” BMI was between 18.5 and 24.9 kg/m²; the “overweight” range was between 25.0 and 29.9 kg/m²; and “obesity” was characterized by a BMI of 30 kg/m² or higher [29].

- Vegetarian and Non-Vegetarian

The dietary pattern of the participants was self-reported using the following question: *“What type of diet do you follow?”*, and were classified as non-vegetarians (those who reported consuming red meat and derivatives, poultry, fish, and vegetables) and vegetarians (those who reported consuming milk, eggs, derivatives, and vegetables) [30,31].

### 2.4. Statistical Analysis

Statistical analysis was performed with the statistical program R version 4. Descriptive analysis employed relative and absolute frequencies for categorical variables and mean and standard deviation for numerical variables. Sociodemographic data, depression, anxiety, emotional eating, and BMI of participants were compared based on their dietary pattern. Statistically significant differences in the dietary pattern were considered using the Student’s *t*-test and the chi-squared test of independence. Finally, linear regression (beta) and Poisson regression (prevalence ratios) models with robust variance were used to investigate the association between dietary pattern, depression, anxiety, emotional eating, and BMI. A threshold for statistical significance was established at a *p*-value of less than 0.05 for all tests conducted.

## 3. Results

The sociodemographic data for the vegetarians and non-vegetarians are presented in Table 1. A total of 768 individuals voluntarily decided to participate in this study, of which 37% (*n* = 284) identified themselves as vegetarians and 63% (*n* = 484) as non-vegetarians. The mean age of the vegetarians was significantly lower (33 ± 5.7 years) compared to that of the non-vegetarians (35 ± 7.3 years) (*p* < 0.001). This study found that the vegetarians were significantly younger and more likely to be female, from coastal areas of the country, Seventh-day Adventists, educated at the postgraduate level, and have an income range of 2149.00–10,746.00 compared to the non-vegetarians (*p* < 0.05).

Table 2 compares the characteristics related to the mental health and BMI of the participants. Statistically significant differences were found in the mean BMI score between the vegetarians (23.2 ± 3.2) and non-vegetarians (24.1 ± 3.2), *p* = 0.011; furthermore, this study found that a higher percentage of non-vegetarians (32.9%) were classified as overweight or obese compared to the vegetarians (27.9%; *p* < 0.001). This study found that the mean depression score was significantly higher in the vegetarians (2.82 ± 2.74) than in the non-vegetarians (1.88 ± 2.36; *p* < 0.001). Furthermore, 26.8% of the vegetarians reported symptoms of depression (score ≥ 3), compared to 18.9% of the non-vegetarians, *p* = 0.038. Similarly, this study found that the mean anxiety score was higher in the vegetarians (2.02 ± 2.12) compared to the non-vegetarians (1.65 ± 0.98), *p* = 0.023. This study found that the vegetarians (32.6%) reported higher levels of anxiety symptoms (score ≥ 3) compared to the non-vegetarians (26.9%), with a statistically significant difference (*p* < 0.001). However, it was found that the non-vegetarians tend to engage in emotional eating more frequently.

Table 3 presents the results of the simple and multiple regression analyses that examined the association between the dietary pattern (vegetarian vs. non-vegetarian) and depression, anxiety, emotional eating, and BMI. The vegetarians (adjusted PR = 0.24, 95% CI: 0.16–0.31; *p < *0.001) were significantly more likely to exhibit depressive symptoms compared to the non-vegetarians. This trend was maintained for anxiety, with an adjusted PR = 0.17, 95% CI: 0.01–0.29; *p* = 0.012. However, the vegetarians (adjusted PR = −0.38, 95% CI: −0.61–−0.14; *p* < 0.001) reported significantly lower EmE scores than the non-vegetarians. Furthermore, the vegetarians had a significantly lower mean BMI than the non-vegetarians (B = −0.16, 95% CI: −0.21–−0.08; *p* < 0.001).

## 4. Discussion

The relationship between diet and mental health has been a topic of growing interest in the scientific literature, recognizing diet as not only a determinant of physical health but also of psychological well-being. The appearance of health problems, including depression, anxiety, and obesity, as well as maladaptive eating behaviors, raises significant public health concerns. This cross-sectional study examines depression, anxiety, EmE, and BMI among Peruvian adults following vegetarian and non-vegetarian diets. The main findings indicated that (a) compared to the non-vegetarians, the vegetarians exhibited a significantly higher likelihood of experiencing symptoms of depression; (b) in addition, the vegetarians were significantly more likely to report anxiety symptoms compared to the non-vegetarians; (c) however, the vegetarians exhibited a lower tendency toward emotional eating compared to the non-vegetarians; finally, it was found that the vegetarians had a significantly lower average BMI than the non-vegetarians.

- Depression

Depression, as a clinical and subjective phenomenon, can manifest itself in a variety of ways and its prevalence and severity can be influenced by multiple factors, including dietary patterns [32]. The data from the current study suggest that the vegetarians reported higher scores on the depression measures compared to the non-vegetarians. These findings are consistent with other similar studies that have reported an association between vegetarian diets and a higher prevalence of depressive symptoms [7,33]. There are several reasons that could be behind this association. On the one hand, people who are already experiencing depression may be drawn to vegetarian diets, possibly due to the belief that altering their diet can improve their health and well-being; however, further exploration is needed to better understand the temporal relationship between the onset of depression and the adoption of this eating behavior [34].

However, vegetarians may face unique stressors related to their dietary choice [35]. These difficulties may include finding suitable food options and a lack of social support [35]; for instance, in Western societies, vegetarians, who are often a minority, may encounter prejudice due to their dietary choices. These attitudes may have a detrimental effect on their psychosocial well-being, which could worsen feelings of isolation or social exclusion [34]. In fact, demographic studies from Asian countries, where vegetarianism is more prevalent, and among communities practicing religions with strong vegetarian traditions, do not suggest a significant association between vegetarianism and depression [13,36]. This discrepancy suggests that the cultural acceptance of vegetarianism may moderate its relationship with mental health [37]. Thus, the varying degrees of acceptance or stigmatization of vegetarianism in different cultural contexts could explain the variability in the psychological effects of this dietary pattern. Another possible explanation is that the emotional burden of ethical or environmental concerns often motivates vegetarianism [38,39], these factors may contribute to a higher level of psychological stress, which could worsen or present as symptoms of depression.

However, it is important to note that research on the association between vegetarian diets and depressive symptoms is ambiguous and lacks consensus. For example, while the findings of some systematic reviews have not revealed an association between vegetarian diets and depressive symptoms [4,40], other studies suggest that following a vegetarian diet is associated with a lower risk of depression [41,42,43]. Likewise, findings from a cross-sectional study conducted among members of the Seventh-day Adventist Church (SDA) who consumed a vegetarian diet showed significantly fewer negative emotions compared to non-vegetarians [13]. The difference in mental health observed among vegetarian Adventists may be attributed to the healthy lifestyle promoted by the SDA Church, which includes abstinence from tobacco and alcohol, physical activity, adequate rest, and strong community ties and social support [44,45]. On the other hand, a potential underlying biological mechanism is that healthy dietary interventions, particularly those rich in plant-based foods, reduce systemic inflammation and inflammatory biomarkers [46,47], which have shown a correlation with depressive symptoms [48]. In parallel, it has been documented that depression is accompanied by increased oxidative stress and inflammation at the brain level [48]. In this context, it has been observed that vegetarian diets, which are high in antioxidant compounds such as polyphenols, have a negative correlation with the incidence [49,50] and severity of depression [51].

- Anxiety

Anxiety is a prevalent mental disorder globally [9], and recently, there has been growing interest in the role of eating habits as a risk factor for this psychiatric disorder [52]. In the present study, it was observed that vegetarians had significantly higher levels of anxiety compared to their non-vegetarian counterparts. This observation aligns with previous research, which has found evidence linking vegetarian diets to higher rates of anxiety disorders [6,53,54]. For instance, a recent meta-analysis of around 170,000 participants concluded that people who consume meat experience less anxiety than those following a plant-based diet [55].

One of the most prominent hypotheses for this association is that nutritional deficiencies, commonly associated with restrictive vegetarian diets, such as low intakes of omega-3 fatty acids, zinc, and vitamin B-12, can contribute to mood disturbance [6,7,8]. For example, one study reported very low levels of omega-3 polyunsaturated fatty acids in vegetarians and vegans [56]. This reduction in omega-3 polyunsaturated fatty acid intake among some vegetarians may be due, in part, to the fact that the primary bioavailable source of these fatty acids is oily fish [57]. Deficiencies in these fatty acids could have a negative impact on mental health, brain function, and the modulation of neurotransmitters responsible for mood; this, in turn, could lead to anxiety [58]. Similarly, research has shown that 52% of people following a vegan diet and 7% of those identified as vegetarians had insufficient levels of vitamin B-12 [5], a nutrient typically obtained through the consumption of red meat and considered beneficial in mitigating symptoms of anxiety [59].

An additional explanation for the elevated levels of anxiety in vegetarians may lie in psychosocial factors that can affect mental well-being [60,61,62]. This sensitivity to the environmental and ethical impacts of food choices can lead to what is commonly known as “ecological anxiety” or “climate anxiety” [61]. This form of distress is characterized by experiencing intense negative emotions, such as fear, worry, guilt, shame, hopelessness, and despair, in response to contemporary environmental challenges [62]. Such emotional states, when experienced in a prolonged or intense manner, can intensify anxiety levels, suggesting a profound connection between environmental concerns and the mental health of vegetarian individuals.

However, the literature on vegetarian diets and anxiety symptoms is controversial. For example, findings from a systematic review and meta-analysis found no significant association between the vegetarian diet and anxiety [63]. Similarly, other studies have found no association between plant-based diets and anxiety, even after adjusting for relevant factors [22]. In addition, one study reported that vegetarianism is not associated with mental health in the US, Russia, or Germany; however, it is associated with slight increases in anxiety in college students in China [64]. On the other hand, an unhealthy vegetarian diet, characterized by excessive consumption of processed and refined foods, rich in added sugars and saturated fats but poor in essential nutrients, is associated with an increased risk of anxiety. In contrast, a healthy vegetarian diet, which emphasizes the intake of whole foods such as fruits, vegetables, legumes, whole grains, nuts, and seeds, offering a diversity of vitamins, minerals, fiber, protein, and healthy fatty acids, is not associated with anxiety [65]. It is suggested that vegetarian diets may have a protective effect against anxiety when planned and followed in a nutritionally adequate manner; this highlights the importance of food quality in vegetarianism and its impact on mental health, indicating that the nutritional composition of the diet, in addition to the dietary pattern, is crucial for psychological well-being. This complements other research indicating that people who follow a predominantly plant-based diet experience reduced levels of anxiety compared to those who maintain omnivorous diets [42]. In accordance with this point, some studies suggest that vegetarian diets, which are rich in plant-based products, offer numerous health benefits, including positive effects on mood and psychological well-being due to the variety of essential nutrients present in them that promote mental health [46,66].

- EmE

Emotional eating is described as the tendency to consume excessive food in response to negative emotions such as emotional stress, anxiety, frustration, sadness, anger, and loneliness [18,67]. This study found that vegetarians are less likely to engage in emotional eating compared to non-vegetarians. In a similar study, vegetarians had the lowest scores on the subscales measuring emotional eating [17]. In another study, it was observed that vegans reported a lower tendency to eat emotionally [18], which is consistent with our findings. Similarly, a study evaluating restrained eating and dietary intake in vegans, vegetarians, and omnivores found that the vegetarian and vegan groups reported a lower tendency towards emotional eating compared to those following an omnivorous diet [67]. This could be interpreted to mean that non-vegetarians have developed a stronger association between food consumption and mood fluctuations, possibly resorting to food as a coping mechanism in times of emotional distress [68]. In our explanation, it is also possible that vegetarian diets tend to be more structured and may require greater awareness and meal planning, which could contribute to a more reflective relationship with food and less dependence on food as an emotional coping mechanism.

However, the literature on the relationship between a vegetarian diet and emotional eating behaviors is not unanimous, being equivocal. Some studies indicate that vegetarians can display maladaptive eating behaviors, which could suggest a tendency toward problematic eating behaviors [1,7,69]. It has been suggested that vegetarianism may be used as a cover for eating disorders, allowing those affected to have a socially acceptable excuse to reject certain food groups or avoid specific food situations [17]. Furthermore, adopting a vegetarian diet can increase the risk of developing eating disorders in individuals with certain predisposing personality traits [70]. Therefore, the investigation of a direct causal link between vegetarianism and eating disorders is still an ongoing research topic.

- BMI

Finally, in our study, the linear regression analysis showed that the vegetarians were less likely to have excess body weight, which was also reflected in the lower BMI scores. These findings support recent research indicating that vegetarian diets are associated with a lower BMI and a reduced risk of obesity [71,72]. For instance, studies such as that by Turner-McGrievy et al. [71] and the systematic review by Huang et al. [72] demonstrate the effectiveness of a vegetarian diet in maintaining a healthy weight. It is suggested that vegetarian diets, due to their lower calorie density and higher fiber content, promote satiety and can result in a lower overall calorie intake and subsequently lower body weight [2,71].

Furthermore, adhering to a vegetarian diet can indicate increased awareness and concern for personal health and well-being, potentially leading to increased efforts to maintain a healthy weight [73]. Similarly, it is possible that our findings are at least partially attributable to the lower likelihood of emotional eating among the vegetarians compared to the non-vegetarians. This observation is crucial because emotional eating has often been associated with a higher caloric intake, increasing the risk of overweight and obesity [15]. The lower incidence of emotional eating among the vegetarians suggests a possible mechanism to maintain a lower BMI. On the other hand, it is also important to consider that vegetarian and vegan diets may not provide sufficient high-quality protein, essential vitamins such as B-12, and minerals such as iron and zinc. This could lead to a decrease in muscle mass, which could affect BMI [74,75]. Therefore, when considering the lower BMI among the vegetarians and vegans, it is important to distinguish between healthy weight loss and the potentially detrimental loss of muscle mass. This reinforces the importance of careful dietary planning and nutritional counseling for individuals who choose to follow plant-based diets, ensuring that these diets are nutritionally complete and support both metabolic health and optimal body composition. In any case, our study contributes to the existing body of knowledge, highlighting the complexity of dietary and behavioral factors in maintaining a healthy weight, and highlights the importance of future research to explore these links in more depth and over time.

### 4.1. Limitations and Future Research

This study has several limitations that should be considered when interpreting the results. Although our findings suggest an association between a vegetarian diet and a higher probability of anxiety and depressive symptoms, as well as a lower risk of emotional eating and BMI, it is important to note that cross-sectional data cannot establish the causality or direction of the relationships between these variables. Another important limitation to consider is that an individual’s choice and motivation to follow a particular diet may be influenced by their previous mental health status. It is possible that people may choose to adopt a vegetarian diet after experiencing a mental disorder, in the hope of improving their condition [6,33]. This hypothesis proposes a multifaceted correlation between mental health and dietary preferences that warrants further investigation [7]. In addition, the weight and height data of the participants were self-reported. Previous research has confirmed the reliability of self-assessments when compared to direct measurements; however, there are still notable differences when these data are compared with objective measurement methods [76]. A third limitation of this study is that the majority (85.9%) of the vegetarian participants are affiliated with the SDA Church. This community promotes healthy lifestyles, including physical activity and specific diets, such as vegetarian diets, which can have an impact on the mental and physical health outcomes of its members [13]; also, this may not be representative of all vegetarians and could affect the generalizability of the results. In addition, the participants were recruited by convenience sampling, which may introduce selection bias. On the other hand, this study did not consider potential confounding factors that may affect the relationship between vegetarianism and mental health outcomes, such as lifestyle factors and comorbidities. Furthermore, this study did not include an assessment of the nutritional adequacy of the vegetarian and non-vegetarian diets. It is important to consider the nutritional composition and quality of these diets as they may have a significant impact on mental health outcomes. Finally, the retrospective analysis of the depression and anxiety indicators, limited to one week, restricts our ability to comprehensively understand these conditions throughout the life course of the study subjects. A detailed clinical review of the history of these conditions or data on previous incidents are necessary to gain a more complete understanding [34].

To clarify the causality and direction of the relationships between a vegetarian diet and mental health, future research should employ longitudinal designs, considering the limitations of previous studies. In addition, it would be advantageous to incorporate objective measurements of weight and height to further investigate the correlation between dietary motivation and mental health. It is also important to take into account the impact of religious and cultural factors on these connections. Future research should explore these associations in different contexts, such as cultural, social, and environmental, to assess the consistency of the results and better understand the underlying dynamics. Finally, including detailed clinical evaluations of the participants’ mental health history would improve our understanding of the connections between diet and mental health over time.

### 4.2. Implications for Public Health

The results of this study have significant implications for public health, particularly in terms of how mental health and weight management are addressed in both vegetarian and non-vegetarian populations. First, these findings indicate a need for focused psychological evaluation and support for this group. Health professionals, especially nutritionists/dietitians and psychologists, should be aware of these potential vulnerabilities to provide appropriate preventive and therapeutic interventions. This includes nutritional counseling to ensure the adequate intake of essential nutrients that can influence mood, such as omega-3 fatty acids and vitamin B-12. Furthermore, it highlights the importance of addressing this problem by creating stress and anxiety management techniques that are customized to the requirements of vegetarians, taking into account both the dietary and psychosocial aspects of their overall care. Second, the findings suggest an area for intervention in education regarding healthy eating habits and the development of alternative coping strategies to manage negative emotions without resorting to food. Promoting a healthy relationship with food and effective emotional management may be particularly important in preventing the development of eating disorders and weight gain in the general population. Finally, this study highlights the potential of vegetarian diets to aid weight control and reduce the risk of chronic diseases associated with overweight and obesity, such as type 2 diabetes, cardiovascular disease, and certain types of cancer. From a public health perspective, the promotion of plant-based diets could be an effective strategy to combat the obesity epidemic, provided that it is performed in a balanced and nutritionally complete manner.

## 5. Conclusions

This study examines the association between a vegetarian diet and different aspects of mental and physical health, including symptoms of anxiety and depression, emotional eating, and BMI, in an adult population. This study indicates a significant association between the vegetarian diet and higher levels of anxiety and depressive symptoms. In addition, people who follow a vegetarian diet are less likely to engage in EmE and have a lower BMI. These findings highlight the importance of a longer-term integrative approach to the study of dietary patterns and health, focusing on the fact that dietary choices are not only related to physical fitness but also mental health.

## Figures and Tables

**Table 1:** General characteristics of the participants according to the dietary pattern (*n* = 768).

Characteristics	Vegetarians	Non-Vegetarians	*p* ^a^
	(*n* = 284, 37%)	(*n* = 484, 63%)	
Age (M ± SD)	33 ± 5.7	35 ± 7.3	<0.001
Age group			<0.001
18–24	61 (21.4%)	83 (17.1%)	
25–34	114 (40%)	150 (30.9%)	
35–44	58 (20%)	184 (38.1%)	
>44	53 (18.6%)	67 (13.9%)	
Sex			0.034
Female	170 (60%)	233 (48.2%)	
Male	134 (40%)	251 (51.8%)	
Region of origin			0.037
Coast	177 (62.4)	93 (19.2%)	
Highlands	24 (8.4%)	265 (54.7%)	
Jungle	32 (11.2%)	126 (26.1)	
Religion			0.024
Adventist	244 (85.9%)	97 (20%)	
Baptist	14 (5%)	97 (20%)	
Catholic	11 (3.9)	194 (40%)	
Others	15 (5.2%)	97 (20%)	
Marital status			0.454
Married	145 (51.1%)	258 (53.3)	
Single	139 (48.9%)	226 (46.7)	
Education level			0.049
Basic	28 (10%)	60 (12.3%)	
Technical	57 (20%)	97 (20%)	
Undergraduate	114 (40%)	251 (51.8%)	
Postgraduate	85 (30%)	76 (15.9%)	
Monthly family income			<0.001
<2149.00	114 (40%)	251 (51.8%)	
2149.00–10,746.00	142 (50%)	128 (26.5%)	
>10,746.00	28 (10%)	105 (21.7%)	

Note: Mean (M); standard deviation (SD). ^a^ Student’s *t*-test; chi-squared test of independence.

**Table 2:** Mental health status and anthropometric parameters according to the dietary pattern of the participants (*n* = 768).

Characteristics	Vegetarians	Non-Vegetarians	*p* ^a^
	(*n* = 284, 37%)	(*n* = 484, 63%)	
BMI score	23.2 (3.2)	24.1 (3.2)	0.011
Body Mass Index categorized			<0.001
Underweight	7 (2.3%)	13 (2.7%)	
Normal	199 (70.0%)	311 (64.2%)	
Overweight	67 (23.8%)	132 (27.2%)	
Obesity	11 (3.9%)	28 (5.7%)	
Depression score (M ± SD)	2.82 ± 2.74	1.88 ± 2.36	<0.001
Depression			0.038
Depression < 3 *	208 (73.2%)	393 (81.1%)	
Depression ≥ 3 **	76 (26.8%)	91 (18.9%)	
Anxiety score (M ± SD)	2.02 ± 2.12	1.65 ± 0.98	0.023
Anxiety			<0.001
Anxiety < 3 *	191 (67.4%)	354 (73.1%)	
Anxiety ≥ 3 **	93 (32.6%)	132 (26.9%)	
EmE score (M ± SD)	8.65 ± 4.32	10 ± 6.74	<0.001
EmE			<0.001
Non-EmE	59 (20.9%)	55 (11.3%)	
Low EmE	157 (55.3%)	290 (60.0%)	
EmE	49 (17.1%)	105 (21.6%)	
Very EmE	19 (6.7%)	34 (7.1%)	

Note: Mean (M); standard deviation (SD). ^a^ Student’s *t*-test; chi-squared test of independence. * No depressive symptoms and absence of anxiety. ** With depressive symptoms and indicative of anxiety. EmE = Emotional eating.

**Table 3:** Simple and multiple regression model of depression, anxiety, EmE, and BMI according to the dietary pattern.

	Simple Regression			Multiple Regression ^a^		
	Depression					
Dietary pattern	PR	95% CI	*p*	PR	95% CI	*p*
Non-vegetarian	Ref.			Ref.		
Vegetarian	0.23	0.15–0.30	<0.001	0.24	0.16–0.31	<0.001
	Anxiety					
Dietary pattern	PR	95% CI	*p*	PR	95% CI	*p*
Non-vegetarian	Ref.			Ref.		
Vegetarian	0.14	0.02–0.29	0.010	0.17	0.01–0.29	0.012
	EmE					
Dietary pattern	PR	95% CI	*p*	PR	95% CI	*p*
Non-vegetarian	Ref.			Ref.		
Vegetarian	−0.31	−0.49–−0.11	<0.001	−0.38	−0.61–−0.14	<0.001
	BMI					
Dietary pattern	B	95% CI	*p*	B	95% CI	*p*
Non-vegetarian	Ref.			Ref.		
Vegetarian	−0.12	−0.20–−0.01	0.014	−0.16	−0.21–−0.08	<0.001

Note: PR: prevalence ratio; B: regression coefficient; 95% CI: 95% confidence interval; BMI: body mass index. Linear regression was used for the BMI score variable and Poisson regression with robust variance was used for the variables depression, anxiety, and emotional eating. ^a^ Adjusted for age group, sex, household income, and level of education.

## Data Availability

The data presented in this study are available on request from the corresponding author. The data are not publicly available due to privacy and ethical concerns.
